# DMD mutations in pediatric patients with phenotypes of Duchenne/Becker muscular dystrophy

**DOI:** 10.1515/med-2024-0916

**Published:** 2024-11-15

**Authors:** Liping Ge, Yang Yang, Yanfei Yang, Yanfei Chen, Na Tao, Liping Zhang, Canmiao Zhao, Xing Zhang

**Affiliations:** Department of Endosecretory Genetic and Metabolic Diseases, Kunming Children’s Hospital, Kunming 650000, China; The Special Wards, Kunming Children’s Hospital, Kunming 650000, Yunnan Province, China; Department of Cardiovascular Internal Medicine, Kunming Children’s Hospital, Yunnan Province, Kunming 650000, China; Medical Department, Kunming Children’s Hospital, Kunming 650000, China

**Keywords:** *DMD*, dystrophin, next-generation sequencing, DMD/BMD

## Abstract

Duchenne muscular dystrophy (DMD) and Becker muscular dystrophy (BMD) are common X-inherited neuromuscular diseases. The genetic diagnosis has been used as the diagnostic choice for DMD/BMD. The study subjects consisted of 37 patients from Southwest China. Peripheral blood was collected for the extraction of genomic DNA. DMD mutation was sequenced using the next-generation sequencing approach. The detected mutation was validated using the multiplex ligation-dependent probe amplification or Sanger sequencing methods. Variation annotation and pathogenicity prediction were performed using the online databases. Pathogenic mutations were identified 3 splicing site, 7 single nucleotide, 1 indel, 23 deletion, and 3 duplication mutations. Novel DMD variants were discovered, including two novel splicing variations (c.1890 + 1G>T; c.1923 + 1G>A), one missense mutation (c.1946G>T), one nonsense mutation (c.7441G>T), one indel mutation (INDEL EX20), and one duplication mutation (DUP EX75-78). The current study provides mutation information of DMD for the genetic diagnosis of DMD/BMD.

## 1 Introduction

Duchenne muscular dystrophy (DMD; MIM number 310200) is an X-linked recessive condition with a high incidence of new mutations, mostly out-of-frame deletions in the dystrophin gene [1,2]. A milder allelic Becker muscular dystrophy (BMD; MIM number: 300376) is caused by mutations that produce dystrophin with abnormal size [3]. DMD and BMD are defined as progressive deterioration of muscle tissue and resultant weakness [4,5]. Patients with DMD first showed apparent symptoms at the age of 2–3 years. Due to the gradual loss of muscle tissue, wheelchair dependence occurs at around the age of 12 years. Ventilation assistance had a median age of 20 years. Patients with BMD have similar signs and symptoms to those with DMD, except that the time course is more varied, and the onset is later. The standard of care for DMD and BMD integrates a multidisciplinary strategy that includes ventilation and coughing assistance, gastrointestinal tube feeding, cardiomyopathy treatment, and delaying loss of ambulation through corticosteroids [6,7]. However, all these therapeutic approaches only slow down the progression of the disease, not restoring the expression and function of dystrophin proteins.

The dystrophin gene (*DMD*; NCBI Gene LOCUS: NG_012232; NCBI Gene ID: 1756) is located at locus Xp21.2, spanning more than 2,200 kb and consisting of 89 exons. The primary dystrophin isoform is full-length Dp427 (UniProt Isoform ID: Dp427m, P11532-1; Dp427c, P11532-4; Dp427p, P11532-11) mainly expressed in skeletal and cardiac muscles and detected in the central nervous system [8,9]. Dp116 (UniProt Isoform ID: P11532-17) is expressed in peripheral nerves, Dp71 (UniProt Isoform ID: Dp71, P11532-7; Dp71a, P11532-8; Dp71b, P11532-6; Dp71ab, P11532-5) is detected in non-muscular tissues, Dp260 (UniProt Isoform ID: Dp260-1, P11532-2; Dp260-2, P11532-3) is mostly expressed in the retina, and Dp140 (UniProt Isoform ID: Dp140, P11532-12; Dp140c, P11532-13; Dp140b, P11532-14; Dp140ab, P11532-15; Dp140bc, P11532-16) is distributed in the kidney, retina, and brain [8,9]. Dystrophin protein functions mainly independent of four domains, containing an actin-binding amino-terminal domain, a central rod domain, a cysteine-rich domain, and a carboxyl-terminus [10,11]. Premature stop codons caused by point mutations or deletions produce truncated protein or elicit undetectable protein expression [12]. The protein domain is essential for dystrophin function, and the loss of the protein domain results in severe phenotypes [13].

It has been postulated that the majority of *DMD* mutations or full-length dystrophin affects spermatogenesis or oogenesis [14,15,16]. *DMD* gene mutations result in clinical phenotypes including severe DMD, BMD, X-linked dilated cardiomyopathy-3B (CMD3B) [3,17]. Out-of-frame dystrophin gene mutations lead to DMD phenotype characteristic of a severe decline or absence of dystrophin in the muscle, while the expression of a partly functional truncated protein was caused by in-frame mutations, causing the milder BMD [18,19]. Duplications have been shown to have a role in cellular communication by acting as a transmembrane signaling complex [20,21]. In this study, we presented 37 patients with rare DMD and investigated the mutations of DMD gene in patients with DMD. Seven novel X-linked genetic variations in *DMD* were described, which may help clinicians to diagnose DMD based on genetic variations.

## 2 Materials and methods

### 2.1 Patient cohort

All consecutive pediatric patients admitted to the Kunming Children’s Hospital (Kunming, Yunnan, China) with analogous symptoms of DMD, BMD, and CMD3B between November 2017 and June 2020 were considered for enrollment in this study. Diagnosis of DMD, BMD, and CMD3B was made according to clinical criteria including infants with ambulation delay, children-teenagers with loss of ambulation, pseudohypertrophic calves, Gowers’ sign, electromyographic pattern concordant with DMD/BMD/CMD3B, and creatine kinase (CK) higher than 3,000 U/L. A total of 37 patients and their families have been referred in this study for molecular analysis of *DMD*. Electromyographic characterizations were recorded using a Nicolet Viking IV EMG machine (VIASYS Healthcare, Madison, Wisconsin).

### 2.2 Blood sample collection and DNA extraction

A total of 3 mL peripheral blood was collected from each individual and stored in ethylene diamine tetraacetic acid tubes. Genomic DNA from peripheral blood was extracted using a QIAamp DNA Blood Mini Kit (Catalogue: 51104) (QIAGEN, Hilden, Germany). The extracted DNA was preserved at −20°C for mutation analysis.

### 2.3 Targeted NGS analysis

After extraction, 500 ng of genomic DNA was sheared into 200-bp fragments using a Covris-S220 ultrasound system (Covaris, Woburn, MA, USA). NEBNext DNA Ultra II reagents (Catalogue: E7645S) (NEB, Ipswich, MA, USA) were used for end repairing, A-tailing, and adaptor ligation according to the manufacturer’s instructions. After modification with indexing-specific adaptors, adaptor-modified ends were purified with AMPure XP beads (Catalogue: A63881) (Beckman Coulter, Brea, CA, USA). PCR amplification was performed for library construction using self-developed DNA standard library preparation kit. Human exon sequences from DNA extract were captured using SureSelect^XT^ Human All Exon V6 Kit (Catalogue: G9704K) (Agilent, Beijing, China). AMPure XP beads were used to purify the enriched products. DNA library was checked with Qubit and 2100 Bioanalyzer (Agilent). Then, the libraries were sequenced by the Illumina NovaSeq 6000 platform (Illumina, San Diego, CA, USA). The reads were aligned with the human genome reference sequence GRCh37/hg19. Variations were confirmed using GATK v.4.0.0.0 (https://gatk.broadinstitute.org/hc/en-us/sections/360007407851-4-0-0-0) and SAM tools (https://sourceforge.net/projects/samtools/files/samtools/1.8/).

### 2.4 Multiplex ligation-dependent probe amplification (MLPA) analysis

MLPA method was used for mutation validation. SALSA MLPA probe sets (P034 and P035) (MRC-Holland, Amsterdam, the Netherlands) were used for screening *DMD* exons according to the manufacturer’s instructions. Then, amplified products were separated using a 3500 XL Genetic Analyzer (ABI, Carlsbad, CA, USA). Coffalyser software (MRC-Holland) was used to analyze the data.

### 2.5 Sanger sequencing

Sanger sequencing was performed to confirm the variants detected by targeted NGS or MLPA. PCR amplification was done using TaKaRa LA PCR™ Kit (Catalogue: RR013A) (TaKaRa, Dalian, China) and specific primers for *DMD*. After purification with NucleoSpin Gel and PCR clean-up (Catalogue: 740609.50) (MACHEREY-NAGEL, Duren, Germany), the products were sequenced using BigDye Terminator v3.1 Cycle Sequencing Kit (Catalogue: 43-374-58) (ABI). The extension products were diluted using Hi-Di formamide (Catalogue: 4311320) (Applied Biosystems). After denaturalization (95°C, 5 min), DNA products were sequenced on ABI 3500XL DNA Analyzer (ABI). The raw sequence data reported in this article have been deposited in the Genome Sequence Archive (Genomics Proteomics & Bioinformatics 2021) in National Genomics Data Center, China National Center for Bioinformation/Beijing Institute of Genomics, Chinese Academy of Sciences (GSA-Human:HRA005238), that are publicly accessible at https://ngdc.cncb.ac.cn/gsa-human/s/sY4sdKbI [22,23].

### 2.6 Variation annotation, pathogenicity, and pathogenic evaluation

The screened variants were annotated based on publicly available databases including the Human Gene Mutation Database (HGMD) (http://www.hgmd.cf.ac.uk/ac/index.php), ClinVar (https://www.ncbi.nlm.nih.gov/clinvar/), ClinGen (https://www.clinicalgenome.org/), Genome Aggregation Database (https://gnomad.broadinstitute.org/), dbSNP (https://www.ncbi.nlm.nih.gov/projects/SNP/), and The International Genome Sample Resource (https://www.internationalgenome.org/). Variation pathogenicity was predicted using SIFT [24], PolyPhen_2 [25], and REVEL [26]. The pathogenic analysis of the variants was performed according to the guidelines issued by the American College of Medical Genetics and Genomics (ACMG) [27].

### 2.7 Structure prediction

eDystrophin Website (http://edystrophin.genouest.org/) was used for establishing homology models. I-TASSER (https://zhanggroup.org//I-TASSER/) was used to predict protein structure and function.

### 2.8 Patient and public involvemen*t*

Patients and/or the public were not involved in the design, or conduct, or reporting, or dissemination plans of this research.


**Ethical approval:** The research related to human use has been complied with all the relevant national regulations, institutional policies and in accordance with the tenets of the Helsinki Declaration, and has been approved by the Ethics Committee of Kunming Children’s Hospital (2022-03-280-K01).
**Informed consent:** The need for the patient informed consent was waived as retrospective anonymized data were used in this study.

## 3 Results

### 3.1 Patient characteristics

The enrolled patients less than 8 years old had progressive proximal muscular dystrophy and pseudohypertrophy of the calves. The level of CKs in the blood was elevated. Serum levels of CK-muscle/brain (CK-MB), lactate dehydrogenase (LDH), LDH isoenzyme 1, hydroxybutyrate dehydrogenase, alanine aminotransferase, and aspartate aminotransferase were shown in Table 1. Electromyography indicated myopathic changes. Muscle biopsy revealed myofiber degeneration showing fibrosis and fatty infiltration.

**Table 1:** The laboratory features of patients with DMD/BMD

Patient no.	CK	CK-MB	LDH	LD1	HBDH	ALT	AST
18C062297	2,504	113	680	163	521	73	79
19C024016	7,310	281	606	90	419	131	121
19C017681	2,889	125	584	109	425	102	67
18C062137	8,292	438	941	119	620	166	194
18C007254	1,368	49	256	56	177	17	43
19C017624	7,753	571	1417	205	1,076	245	253
18C062294	4,776	298	723	90	467	124	135
19C017684	10,461	453	1,130	189	860	302	204
18C053835	18,042	811	1,571	132	1,060	711	337
18C007192	23,210	812	1,468	149	979	566	379
18C047970	9,382	351	813	129	576	222	256
19C088521	907	66	464	107	352	44	58
19C041652	9,360	267	746	69	390	127	182
18C053914	11,456	494	1,067	124	745	402	193
17C053395	2,230	117	526	108	379	75	80
17C066617	2,446	125	483	115	371	87	112
19C088520	1,889	154	783	178	575	82	115
18C047940	13,221	593	1,084	182	786	377	207
17C065493	6,850	235	626	77	383	159	197
18C053873	2,425	128	573	145	458	129	77
18C007049	24,330	826	1,517	185	1,056	374	277
18C056120	11,544	381	1,112	123	750	369	377
18C047958	18,918	911	1,550	183	1,070	531	353
18C047839	4,239	372	982	160	731	235	125
18C066470	896	53	393	108	303	28	54

DMD/BMD, Duchenne/Becker muscular dystrophy; CK, creatine kinase; CK-MB, creatine kinase-muscle/brain; LDH, lactate dehydrogenase; LD1, lactate dehydrogenase isoenzyme 1; HBDH, hydroxybutyrate dehydrogenase; ALT, alanine aminotransferase; AST, aspartate aminotransferase.

### 3.2 DMD mutation analysis

**Figure 1:**
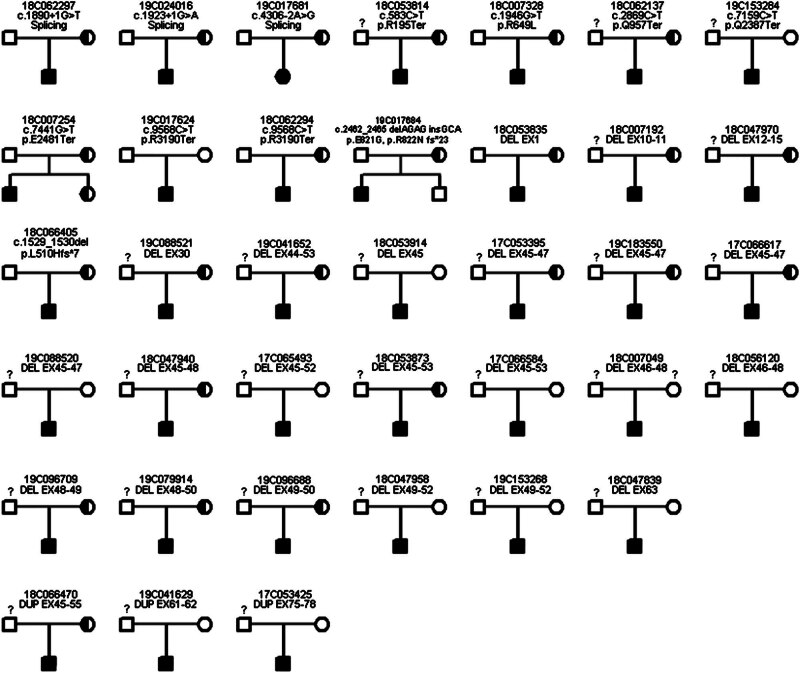
Pedigree of the Chinese family. The fully filled symbol in black indicates patients diagnosed with DMD. The open symbol indicates those unaffected members. The filled semicircle represents asymptomatic carriers. Squares represent males, circles represent females, and the question marks represent individuals that were not sequenced for DMD mutations.

Targeted NGS analysis detected 3 splicing mutations, 7 single nucleotide variations, 1 indel variation, 23 deletions, and 3 duplications, which were confirmed by Sanger sequencing and MLPA (Attached File 1). c.1890 + 1G>T, c.1923 + 1G>A, c.1946G>T (p.R649L), c.7441G>T (p.E2481Ter), c.2462_2465 delAGAG insGCA (p.E821G, p.R822N fs*23), and DUP EX75-78 variations were not reported previously. c.1890 + 1G>T and c.1923 + 1G>A variations caused the change of splicing site. c.7441G>T (p.E2481Ter) contributed to nonsense variation. c.2462_2465 delAGAG insGCA (p.E821G, p.R822N fs*23) and DUP EX75-78 caused frameshift variations (Attached File 1). DMD mutations in 37 families showed that it was compatible with the X-linked recessive inheritance (Figure 1).

### 3.3 DMD pathogenic evaluation

According to the ACMG guideline, c.1890 + 1G>T was predicted to be likely pathogenic (PVS, PM2). The pathogenic analysis revealed that c.1923 + 1G>A was likely pathogenic (PVS1, PM2). c.1946G>T (p.R649L, a novel variant) and c.7441G>T (p.E2481Ter) (PVS, PM2) were likely pathogenic. DUP EX75-78 was pathogenic (Additional File 1).

### 3.4 Structure and function analysis

**Figure 2:**
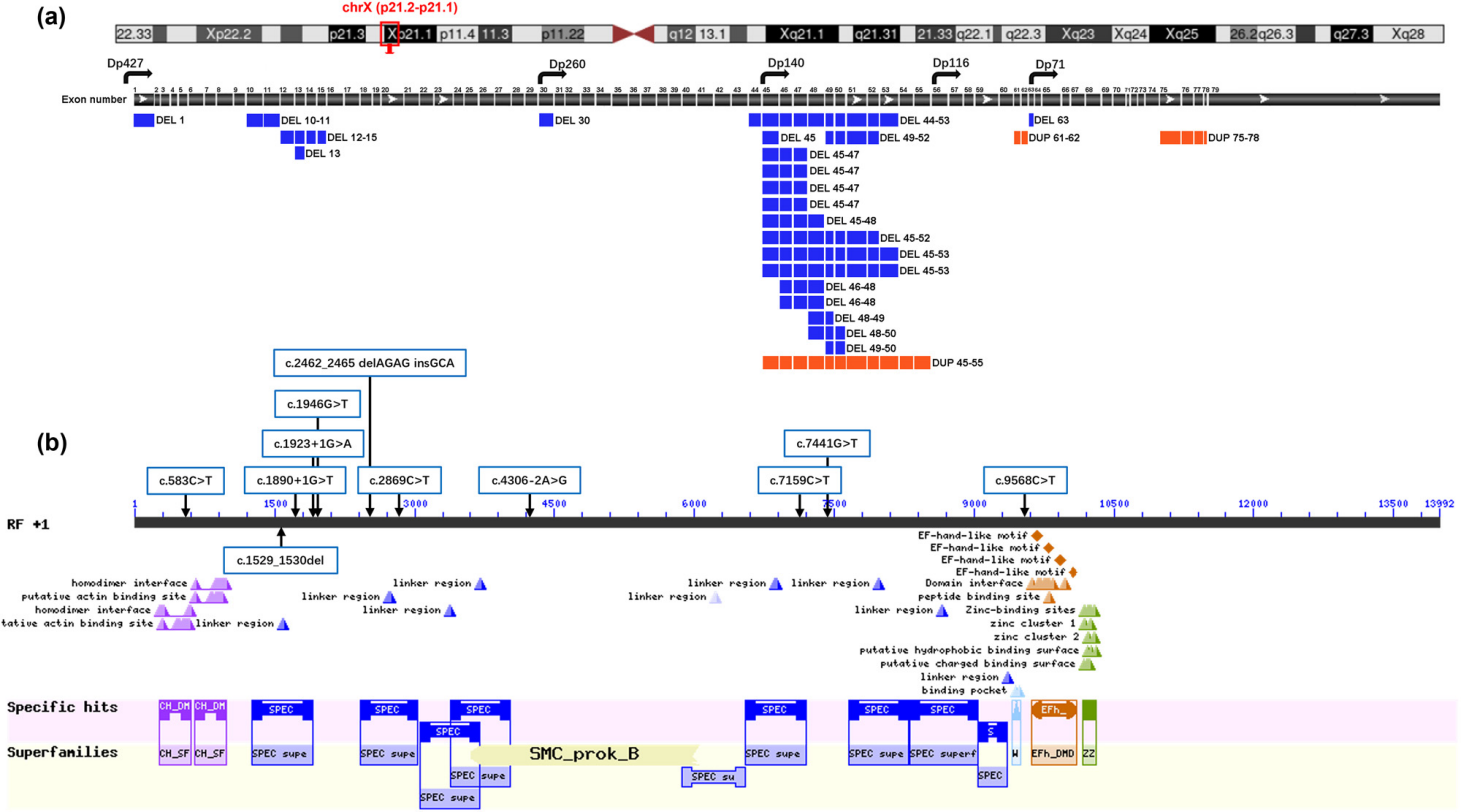
Structure of the DMD, domain structure of dystrophin, and mutation location in this study. (a) Genomic organization of the DMD gene Dp427, cortical neurons, skeletal and cardiac muscles, and Purkinje cells; Dp260, brain, retina layer, and heart; Dp140, brain, retina, and kidneys; Dp116, Schwann cells; Dp71, brain, liver, heart, and retina. (b) Conserved domains of dystrophin protein. EFh_DMD, EF-hand-like motif found in dystrophin; SPEC, spectrin repeats; CH_SF, calponin homology domain found in dystrophin and similar proteins; ZZ, zinc finger; SMC, structural maintenance of chromosomes proteins; WW domains, two conserved tryptophan domains.

Deletion of exon 1, exons 10–11, exons 12–15, exon 13, exon 30, exons 44–53, exon 45, exons 49–52, exons 45–47, exons 45–48, exons 45–52, exons 45–53, exons 46–48, exons 48–49, exons 48–50, exons 49–50, and exon 63 and duplication of exons 45–55, exons 61–62, and exons 75–78 caused the mutation of Dp427m (Figure 2a). Dp260 expression was changed by the deletion of exon 30, exons 44–53, exon 45, exons 49–52, exons 45–47, exons 45–48, exons 45–52, exons 45–53, exons 46–48, exons 48–49, exons 48–50, exons 49–50, and exon 63 and duplication of exons 45–55, exons 61–62, and exons 75–78. Dp140 expression was affected by the deletion of exons 44–53, exons 45, exons 49–52, exons 45–47, exons 45–48, exons 45–52, exons 45–53, exons 46–48, exons 48–49, exons 48–50, exons 49–50, and exon 63 and duplication of exons 45–55, exons 61–62, and exons 75–78. The isoform Dp116 was changed by the duplication of exons 61–62 and exons 75–78 and the deletion of exon 63. DMD phenotypes may be associated with the change of Dp71 expression caused by the deletion of exon 63 and duplication of 75–78. Figure 2b presents the positions of splicing and single-nucleotide mutations. c.583C>T variant may change homodimer interface and native actin binding site. c.1529_1530del, c.1890 + 1G>T, c.1923 + 1G>A, c.1946G>T (a novel variant), c.2462_2465 del AGAG insGCA, c.7159C>T and c.7441G>T variants changed protein linker region and spectrin repeats. c.9568C>T variant may change domain interface and EF-hand-like motif found in dystrophin. Patients enrolled in this study mostly have a deletion of exons clustered in exons 45–53. Dystrophin mutation caused by the deletion of exons 45–53 may affect the binding properties to dystrophin’s second actin-binding domain [28], lipid-binding domain 2 [29], and neuronal nitric oxide synthase [30].

## 4 Discussion

Pathogenic *DMD* variants cause clinical phenotypes of DMD/BMD [31,32,33]. The use of early and accurate genetic diagnosis is essential to reduce the risk of recurrence of DMD [34]. Our study discovered two novel splicing variations (c.1890 + 1G>T; c.1923 + 1G>A), one missense mutation (c.1946G>T), one nonsense mutation (c.7441G>T), one indel mutation (INDEL EX20), and one duplication mutation (DUP EX75-78). Splice site, missense, nonsense, indel, deletion, and duplication mutations were likely pathogenic or pathogenic according to ACMG guidelines [27].

Patients with DMD/BMD have been found carrying thousands of different mutations in *DMD*. According to the HGMD database, a total of 3578 mutations has been detected in *DMD*, including missense/nonsense (693), splicing (300), regulatory (1), small deletions (433), small insertions (163), small indels (49), gross deletions (1,268), gross insertions/duplications (571), and complex rearrangements (100) types. Deletions and duplications cluster in hotspot regions in DMD, which are located at exons 45–55 and are present in approximately 47% of patients [35]. In this study, deletion of exons 45–53 was the most common mutation in patients with DMD. Deletion mutations in the *DMD* shift the normal transcript reading frame, producing nonfunctional dystrophin protein [36,37]. Patients carrying an in-frame deletion of exons 45–55 in *DMD* were found with slightly decreased skeletal and cardiac muscles [38].

The presence of essential splicing signals is necessary for splice site recognition and the accurate splicing of pre-mRNA [39,40]. Pseudoexon involvement has been found in varieties of dystrophinopathies [41]. We also described two novel hemizygous splicing mutations (c.1890 + 1G>T and c.1923 + 1G>A), which were predicted to be likely pathogenic according to ACMG guideline. c.1890 + 1G>T and c.1923 + 1G>A variants may affect spectrin repeats in dystrophin protein, which disrupts the formation of stable contacts for dimerization involved in cytoskeletal structure [42]. Particularly, splice sites affected by mutations are likely to be missed by genomic DNA strategies because of the positioning of amplification primers covering flanking intron–exon boundaries or inside exons [43]. Pre-mRNA splicing changed by *DMD* point variants may clarify pathogenicity in DMD. Splice site mutations enable the generation of aberrant dystrophin isoforms or severely truncated proteins [44].

Indel mutation c.2462_2465 delAGAG insGCA was noticed within exon 20 in *DMD*, which has not been reported previously. c.2462_2465 delAGAG insGCA was predicted to be likely pathogenic according to the ACMG guideline. This indel mutation may affect the stabilizing contacts associated with dimerization [42]. Deletion of exons 12–15 caused frameshift, which may change the expression of Dp427, Dp260, Dp140, Dp116, and Dp71 and elicit likely pathogenic outcome. Duplication of exons 75–78 caused frameshift that may change the expression of Dp71 and protein function. According to the ACMG guideline, duplication of exons 75–78 is pathogenic for patients with DMD. The deletion and duplication of *DMD* were detected by the MLPA method that shows the ability to recognize rarer mutations and breakpoints of some deletions and duplications, compared with molecular genetic testing. Besides, this technique increases the efficiency of mutation detection in carriers, advancing the ability to provide precise genetic counseling [45].

In fact, we detected multiple variants in DMD patients, which have been provided in Additional File 1. The minor allelic frequency was also provided in Additional File 1. However, whether these mutations are related to DMD has not been confirmed in the study. For example, CPT1A variant (c.1910C>T) found in DMD patients has been reported to be related to carnitine palmitoyltransferase 1A deficiency [46]. The other gene variants and the possible symptoms have been listed in Additional File 2. Effective therapies such as genome-editing are developing to restore the expression of functional dystrophin [47]. For the treatment of DMD patients with confirmed exon skipping, Viltolarsen and Golodirsen, have been approved by U.S. FDA for marketing for DMD patients with confirmed dystrophin gene amenable to exon 53 skipping, eteplirsen for exon 52 skipping, casimersen for exon 45 skipping. Clinical trials have confirmed clinical benefits of the four antisense oligonucleotides, based on phosphorodiamidate morpholino oligomer, on DMD patients [48–51].

## 5 Conclusions

In conclusion, our study reported 37 patients with clinical and genetic diagnoses of DMD. Genetic diagnoses were confirmed by *DMD* mutations: 3 splicing site, 7 single nucleotide, 1 indel, 23 deletion, and 3 duplication mutations. Novel *DMD* variants were first discovered, including two novel splicing variations (c.1890 + 1G>T; c.1923 + 1G>A), one missense mutation (c.1946G>T), one nonsense mutation (c.7441G>T), one indel mutation (INDEL EX20), and one duplication mutation (DUP EX75-78). These cases highlight the diagnostic utility of *DMD* variants with pathogenicity in patients with DMD.

## Supplementary Material

Supplementary Table 1

Supplementary Table 2

Supplementary Table 3
